# Study on the effects of Massa Medicata Fermentata with different formulations on the intestinal microbiota and enzyme activities in mice with spleen deficiency constipation

**DOI:** 10.3389/fcimb.2024.1524327

**Published:** 2025-01-07

**Authors:** Xuejuan Liang, Dan Wan, Xinliang Li, Yanmei Peng, Linglong Chen

**Affiliations:** ^1^ Institute of Innovative Traditional Chinese Medicine, Hunan Academy of Chinese Medicine, Changsha, China; ^2^ Scientific Research Department, Hunan Academy of Chinese Medicine, Changsha, China

**Keywords:** Massa Medicata Fermentata, spleen deficiency constipation, enzyme activity, intestinal microbiota, VIP, 5-HT

## Abstract

**Objective:**

This study aims to explore the therapeutic mechanism of Massa Medicata Fermentata (MMF) with different formulations on spleen deficiency constipation in mice by analyzing gastrointestinal hormones, D-xylose, intestinal microbiota, and intestinal enzyme activities.

**Methods:**

A spleen deficiency constipation model was established using an oral administration of Sennae Folium decoction combined with controlled diet and water intake. After successful model establishment, the mice with spleen deficiency constipation were treated with MMF S1, S2, S3. Following the intervention, serum samples from each group of mice were collected to measure VIP, 5-HT, and D-xylose. Additionally, small intestine contents were analyzed for intestinal enzyme activity and subjected to 16S rRNA high-throughput sequencing.

**Results:**

Mice with spleen deficiency constipation showed significant decreases in body weight and fecal water content. In contrast, the body weight of the CS2 and CS3 groups returned to normal levels, and fecal water content in the CS2 and CS3 groups also returned to normal. The MMF S2 and S3 significantly increased protease and sucrase enzymes levels compared with CM group. Serum D-xylose levels were significantly reduced in the CM and CS2 group. VIP levels increased significantly in the CM group but decreased in the CS2 and CS3 groups. Additionally, 5-HT levels in the CM and CS1 groups decreased significantly, with the CS2 group returning to normal and the CS3 group showing significant increases. 16S rRNA sequencing analysis revealed that all three MMF formulations effectively restored the intestinal microbiota composition in mice. LEfSe analysis identified characteristic microbiota linked to different intervention groups. The CS3 group significantly upregulated the chloroalkane and chloroalkene degradation and vibrio cholerae pathogenic cycle pathways compared to the CM group. *Candidatus_Arthromitus* in the CS3 group and *Psychrobacter* in the CS2 group were positive and negative correlations with 5-HT and VIP, respectively.

**Conclusion:**

The three formulations of MMF significantly alleviated spleen deficiency constipation symptoms by modulating intestinal enzyme activities, D-xylose, VIP, and 5-HT levels, and restoring intestinal microbiota balance. *Psychrobacter* and *Candidatus_Arthromitus* were identified as potential biomarkers for the treatment of spleen deficiency constipation. Different formulations of MMF have different mechanisms of regulating constipation through intestinal microbiota.

## 1 Introduction

Constipation is one of the most common functional bowel disorders, and its prevalence increases with age. Constipation not only significantly impacts the quality of life of patients but also increases the economic burden. It is typically characterized by a reduced frequency of bowel movements (usually fewer than three times a week) and is often accompanied by difficulty in defecation and abdominal discomfort (Zhao et al., 2022). According to traditional Chinese medicine (TCM) theory, although the pathological site of constipation is in the large intestine, it is closely related to dysfunction in organs such as the spleen and stomach. Spleen deficiency constipation primarily results from the spleen’s inability to properly transport and transform nutrients, leading to a lack of body fluids that normally moisten the intestines. This lack of fluids weakens peristalsis, resulting in the stagnation of waste and difficulty in defecation (Zhang et al., 2017; Yu et al., 2022).

D-xylose is an important objective indicator for diagnosing spleen deficiency. It is a five-carbon sugar that is primarily absorbed in the upper small intestine and is generally absent in the bloodstream. D-xylose levels reflect the absorptive function of the small intestine (Lei et al., 2023). Vasoactive intestinal peptide (VIP) is a 28-amino acid polypeptide that functions as both an inhibitory neurotransmitter and a gastrointestinal hormone. It is distributed in the mucosa and myenteric plexus of the gastrointestinal tract. VIP relaxes smooth muscles in the intestines and blood vessels, inhibits gastrointestinal smooth muscle contraction, and regulates peristalsis, while enhancing glandular secretion in the mucosa. Elevated levels of VIP can inhibit secretory activities and peristalsis, leading to chronic constipation (Cong et al., 2019). Additionally, serotonin (5-HT) plays a crucial role in regulating gastrointestinal motility by acting on the enteric nervous system, promoting intestinal movement. Research shows that decreased 5-HT secretion can reduce smooth muscle contraction and peristalsis, contributing to constipation (Crowell, 2004; Tan et al., 2021; Si et al., 2022). Both VIP and 5-HT belong to the brain-gut peptide family and are involved in the development of spleen deficiency constipation via the brain-gut axis (Li et al., 2020; Yi et al., 2023a).

Under normal conditions, the intestinal microbiota remains relatively stable and plays various roles in physiological functions, such as secreting enzymes to aid digestion, stabilizing the immune system, and preventing the proliferation of pathogenic bacteria (Zhang et al., 2024). Most patients with constipation exhibit disturbances in their intestinal microbiota, characterized by an increase in pathogenic bacteria and a decrease in beneficial bacteria. Prolonged constipation may further damage the intestinal mucosal barrier, exacerbating dysbiosis (Dimidi et al., 2017; Fu et al., 2021a; Yi et al., 2023a). Studies have shown that the process of establishing spleen deficiency constipation models not only reduces the richness and abundance of intestinal microbiota species but also alters the composition of the intestinal microbiota (Li et al., 2016).

Massa Medicata Fermentata (MMF) is a traditional Chinese medicine made by fermenting a mixture of flour, wheat bran, Armeniacae Semen Amarum, Vignae Semen, Artemisiae Annuae Herba, Herba Xanthii, and Herba Polygoni Hydropiperis at controlled temperatures. In TCM theory, MMF primarily enters the spleen and stomach meridians, with functions that include invigorating the spleen and stomach, promoting digestion, and harmonizing the middle burner (Yin et al., 2021). It is widely used to treat digestive disorders such as food stagnation, spleen and stomach deficiency, abdominal distention, and pediatric indigestion (Gao et al., 2016; Yin et al., 2021). Recent research has shown that MMF not only enhances digestive function but also improves intestinal health by regulating the intestinal microbiota. In mice with spleen deficiency and food accumulation, the abundance of beneficial bacteria such as *Bacteroides* decreased, while harmful bacteria like *Verrucomicrobia* increased. These changes were reversed after intervention with MMF, and the intestinal microbiota structure in the mice returned to a normal state (Wang et al., 2024). Clinical studies have also found that combining Shenqu Xiaoshi oral liquid with intestinal probiotics can effectively relieve symptoms and improve the overall efficacy in treating functional constipation in children (Mu and Li, 2023).

This study focused on mice with spleen-deficiency constipation to investigate the effects of different formulations of MMF on this condition. The research examined its impact on intestinal enzyme activity, D-xylose, VIP, 5-HT levels, and intestinal microbiota. The aim was to provide theoretical support and experimental evidence for the clinical application of MMF in treating spleen deficiency constipation.

## 2 Materials and methods

### 2.1 Animal husbandry

To eliminate the influence of sex on the intestinal microbiota, only male mice were used in this study (Wu et al., 2022). A total of 50 male SPF-grade Kunming (KM) mice, 4 weeks old and weighing between 18 and 22 g, were purchased from Hunan Silaike Jingda Experimental Animal Co., Ltd. (License number: SCXK (Xiang) 2019-0004). The mice were housed in cages in the barrier environment of the Experimental Animal Center at Hunan University of Chinese Medicine (License number: SYXK (Xiang) 2019-0009), with a controlled temperature of 23-25°C, relative humidity of 50%-70%, and a 12-hour light/dark cycle. The standard maintenance feed for the mice was provided by the Experimental Animal Center at Hunan University of Chinese Medicine, and low-fiber rice (COFCO International) was purchased from Walmart. All animal experiments were approved by the Experimental Animal Ethics Committee of Hunan University of Chinese Medicine, under approval number LL2022060106.

### 2.2 Preparation of experimental drugs

Preparation of Sennae Folium decoction: Weigh 250 g of Sennae Folium, soak in 2.5 L of boiling water for 10 minutes, and then filter. The filtrate is concentrated at 75°C to obtain a decoction with a crude drug concentration of 1 g/mL, which is stored at 4°C for later use (Yi et al., 2023a, Yi et al., 2023b).

Preparation of the three formulations of MMF (S1, S2, S3): The three formulations of MMF were prepared according to the 2022 edition of the “Norms for the Processing of Traditional Chinese Herbal Pieces in Henan Province”. Each formulation consisted of flour, wheat bran, Armeniacae Semen Amarum, Vignae Semen, Artemisiae Annuae Herba, Herba Xanthii, and Herba Polygoni Hydropiperis, in the proportions of 30:50:3:3:2:2:2 for S1, 25:50:1:1:5:5:5 for S2, and 50:50:45:45:10:10:10 for S3. The proportions were converted based on the dried weights of the raw materials. Medicinal materials are weighed according to the prescribed ratios. Armeniacae Semen Amarum and Vignae Semen are ground into coarse powder and mixed evenly with wheat flour and wheat bran. Separately, Herba Polygoni Hydropiperis, Artemisiae Annuae Herba, and Herba Xanthii are decocted with water. The decoction is filtered, and the resulting liquid is concentrated into a thick paste. While still hot, the paste is mixed with the previously prepared medicinal powders. The mixture is then left to naturally ferment at 25–35°C for 24–48 hours until the surface is covered with a yellowish-white or grayish-white fungal coat. Finally, the product is collected, pulverized, and dried to yield MMF.

Preparation of MMF suspension: The prepared MMF S1, S2, and S3 formulations were crushed and passed through a 100-mesh sieve. 9.6 g of the powdered MMF S1, S2, and S3 were each mixed with 64 mL of sterile water to create suspensions with a concentration of 0.15 g/mL. The suspensions were freshly prepared and thoroughly mixed before intragastric administration (Yang Z et al., 2023; Jiang Y. et al., 2023).

### 2.3 Establishment of the spleen deficiency constipation mouse model

The induction of spleen deficiency through bitter and cold purgatives is currently a stable method for establishing spleen deficiency models. Sennae Folium is a bitter and cold herb, and its purgative effects lead to significant fluid loss, resulting in the depletion of Qi and the deterioration of spleen and stomach function, which induces spleen deficiency (Wu et al., 2023; Yi et al., 2023a). Additionally, limiting food and water intake weakens vital energy, further damaging the spleen and stomach. Therefore, a combination of restricted food and water intake was used to establish a spleen deficiency constipation model, a method proven to be both mature and effective (Zhu et al., 2022; Wu et al., 2023; Yi et al., 2023a).

Fifty mice were acclimated for three days with free access to food and water. They were then randomly divided into two groups: a normal control group (CC group, n=10) and a model group (n=40). The model group received 0.4 mL of 1 g/mL Sennae Folium decoction by gavage, twice daily, for 7 days, along with normal access to food and water, to induce spleen deficiency. The CC group received an equivalent volume of sterile water. Starting on day 8, the Sennae Folium decoction administration was discontinued, and the mice were subjected to limited access to water (0.5 hours once daily) and fed 4-8 g of low-fiber rice every other day for 8 days. The CC group continued to have free access to food and water. Constipation was induced by further restricting water and food intake in the spleen deficiency model. The successful establishment of the spleen deficiency model was indicated by symptoms such as dull fur, lethargy, sleepiness, weakness, and weight loss, along with reduced D-xylose levels. If these symptoms persisted, accompanied by small, dry, and pellet-like stools with palpable abdominal masses, the spleen deficiency constipation model was confirmed.

### 2.4 Intervention with MMF

After successfully establishing the model, the mice in the model group were randomly divided into four groups: spleen deficiency constipation group (CM group, n=10), MMF S1 intervention group (CS1 group, n=10), MMF S2 intervention group (CS2 group, n=10), and MMF S3 intervention group (CS3 group, n=10), with 10 mice in each group. Beginning on day 16, the mice in the CS1, CS2, and CS3 groups received intragastric administration of MMF S1, S2, and S3 suspensions at a dose of 4 g/(kg·d), twice daily, for 7 days (Gao et al., 2016; Jiang Y. et al., 2023). The CC and CM groups received an equal volume of sterile water.

### 2.5 General observations, body weight, and fecal water content measurement

The general characteristics of the mice in each group was observed throughout the experiment, including spontaneous activity, mental state, and fecal characteristics. The body weights of the mice were recorded on day 1 (before modeling) and day 22 (after the MMF intervention). The body weight change rate was calculated as follows:


$$\begin{array}{c} Body\;weight\;change\;rate=\\ \frac{Body\;weight\;after\;MMF\;intervention\;-\;Body\;weight\;before\;modeling}{Body\;weight\;before\;modeling}\times 100\% \end{array}$$

Fresh fecal samples were collected after the intervention, weighed to obtain the wet weight, and dried in an oven at 110°C to constant weight to obtain the dry weight. Fecal water content rate was calculated as follows:


$$\begin{array}{c} Fecal\;water\;content\;rate=\\ \frac{fecal\;wet\;weight-fecal\;dry\;weight}{fecal\;wet\;weight}\times 100\% \end{array}$$

### 2.6 Sample collection

At the end of the experiment, mice were anesthetized using isoflurane, and blood samples were collected via retro-orbital bleeding. Blood samples from each group were left at room temperature for 30 minutes and then centrifuged at 3000 rpm for 10 minutes at 4°C to separate the serum, which was stored at -80°C for subsequent ELISA analysis. Under sterile conditions, intestinal contents from the small intestines of 5 mice in each group were collected for enzyme activity assays and stored at -80°C. Additionally, intestinal contents from the remaining 5 mice in each group were collected and stored in sterile EP tubes at -80°C for subsequent 16S rRNA high-throughput sequencing (Shao et al., 2024; Qiao et al., 2024).

### 2.7 Measurement of serum D-xylose, VIP, and 5-HT levels

After the experiment was completed, blood samples were collected from each group of mice, and serum was obtained by centrifugation. The levels of D-xylose, VIP, and 5-HT in the serum samples were measured using an enzyme-linked immunosorbent assay (ELISA). All operations, including sample addition, enzyme addition, incubation, plate washing, color development, reaction termination, and detection, were conducted strictly according to the manufacturer’s instructions. The ELISA kits were provided by Jiangsu Jingmei Biological Technology Co., Ltd.

### 2.8 Measurement of intestinal enzyme activities

Under sterile conditions, distilled water was added to the collected small intestinal contents. The samples were then shaken in a thermostatic shaker for 30 minutes to ensure the complete dissolution of the enzyme proteins. After centrifugation at 3000 rpm for 10 minutes at 4°C, the supernatant was collected (Fang et al., 2024). Protease activity was measured using the Folin-phenol method, starch and sucrase activities were determined using the DNS method, and lactase activity was measured using the ONPG method (Zhou et al., 2023, Zhou et al., 2024). Absorbance values were measured using a UV spectrophotometer, and enzyme activity was expressed in units of U/g per gram of intestinal contents (Guo et al., 2024).

### 2.9 16S rRNA high-throughput sequencing

Five small intestinal content samples from each group were used for 16S rRNA sequencing. DNA extraction, amplification, and library construction were performed by Shanghai Personal Biotechnology Co., Ltd. DNA was extracted using the OMEGA DNA Kit (Omega Bio-Tek Norcross GA USA), following the manufacturer’s instructions. Total DNA was extracted from each cecal content sample and stored at -20°C for further analysis. The quantity and quality of the extracted DNA were assessed using a NanoDrop NC2000 spectrophotometer (Thermo Fisher Scientific) and agarose gel electrophoresis. The bacterial 16S rRNA V3+V4 regions were amplified using specific primers (Forward primer 338F: 5′-ACTCCTACGGGAGGCAGCA-3′; Reverse primer 806R: 5′-GGACTACHVGGGTWTCTAAT-3′). The PCR products were quantified using the Quant-iT PicoGreen dsDNA Assay Kit and sequenced using the Illumina NovaSeq 6000 platform. The raw sequencing data from the small intestinal contents were uploaded to the NCBI database under the accession number PRJNA1087581.

### 2.10 Bioinformatics analysis

(1) The raw sequencing data were denoised to obtain amplicon sequence variants (ASVs). The SILVA database was then used to compare ASV characteristic sequences with reference sequences to assign taxonomic information to each ASV.
(2) Alpha diversity: Alpha diversity refers to the richness and diversity of species in a local, homogeneous environment. Alpha diversity indices, such as Chao1, Observed_species, Shannon, and Simpson, were analyzed using QIIME2 (https://qiime2.org) (Liu et al., 2023).
(3) Beta diversity: Beta diversity analysis examines changes in species composition across different time and spatial scales. Jaccard, Bray-Curtis, unweighted UniFrac, and weighted UniFrac distance matrices were calculated, followed by principal co-ordinates analysis (PCoA) and non-metric multidimensional scaling (NMDS) analyses for visualization using QIIME2 (Ramette, 2007).
(4) LEfSe analysis: LEfSe analysis (http://huttenhower.sph.harvard.edu/lefse/) combines non-parametric Kruskal-Wallis and Wilcoxon rank-sum tests with linear discriminant analysis (LDA) to identify robust differentially abundant species between groups. LEfSe emphasizes discovering marker species between different groups (Segata et al., 2011).
(5) KEGG Functional Prediction: PICRUSt2 2.3.0 was used to predict bacterial metabolic functions by comparing 16S rRNA gene sequences against the KEGG database to predict functional pathways in the intestinal microbiota.
(6) Correlation Analysis: Spearman’s correlation analysis was performed using the R stats package to explore the relationships between characteristic microbiota and D-xylose, VIP, and 5-HT levels.

### 2.11 Statistical analysis

Statistical analysis was performed using SPSS version 22.0 software. All quantitative data were expressed as mean ± standard deviation. If two groups’ data followed a normal distribution and had equal variances, an independent samples t-test was used. For non-normally distributed data, the Mann-Whitney U test was applied. For comparisons involving more than two groups, if the data followed a normal distribution and had equal variances, one-way ANOVA was performed, followed by *post hoc* analysis using the Least Significant Difference test. For non-normally distributed data or unequal variances, the Kruskal-Wallis test was applied, and Dunn’s *post hoc* test was used for pairwise comparisons. A significance level of α = 0.05 was used, with *p*< 0.05 considered statistically significant.

## 3 Results

### 3.1 Effects of Massa Medicata Fermentata with different formulations on general characteristics of mice with spleen deficiency constipation

**Figure 1:**
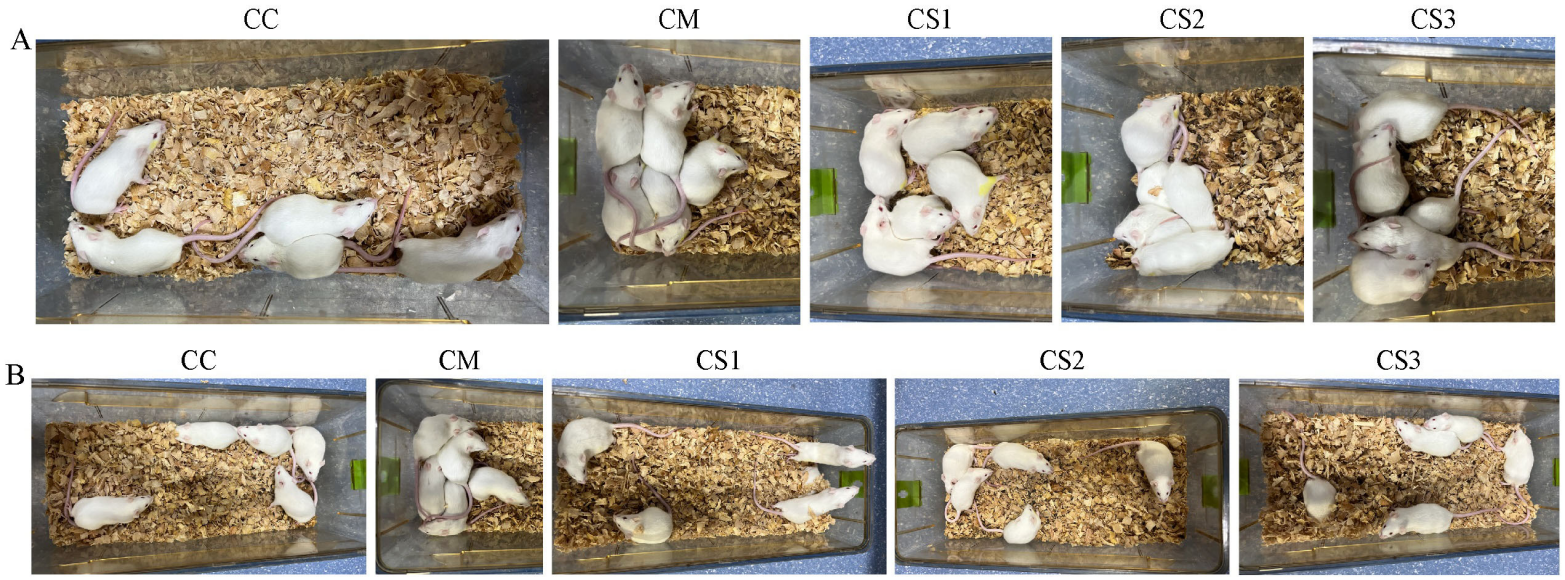
General characteristics of a cage of mice in each group. **(A)** General characteristics during the modeling stage. **(B)** General characteristics after MMF intervention.

As shown in **Figure 1A**, during the modeling stage, mice in the CC group exhibited normal mental states and spontaneous activity, characterized by alertness and a lack of clustering. However, the CM, CS1, CS2, and CS3 groups displayed reduced mental states and spontaneous activity, often clustering together, indicating that the modeling process altered the general characteristics of the mice.

In the treatment stage (**Figure 1B**), mice in the CC group maintained normal mental states and activity, remaining alert without clustering. The CM group continued to show poor mental states and activity, clustering together. In contrast, the CS1, CS2, and CS3 groups gradually recovered normal mental states and activity, showing no clustering behavior. These findings suggest that all three formulations of MMF improved the general characteristics of mice with spleen deficiency constipation.

### 3.2 Effects of Massa Medicata Fermentata with different formulations on body weight and fecal characteristics of mice with spleen deficiency constipation

**Figure 2:**
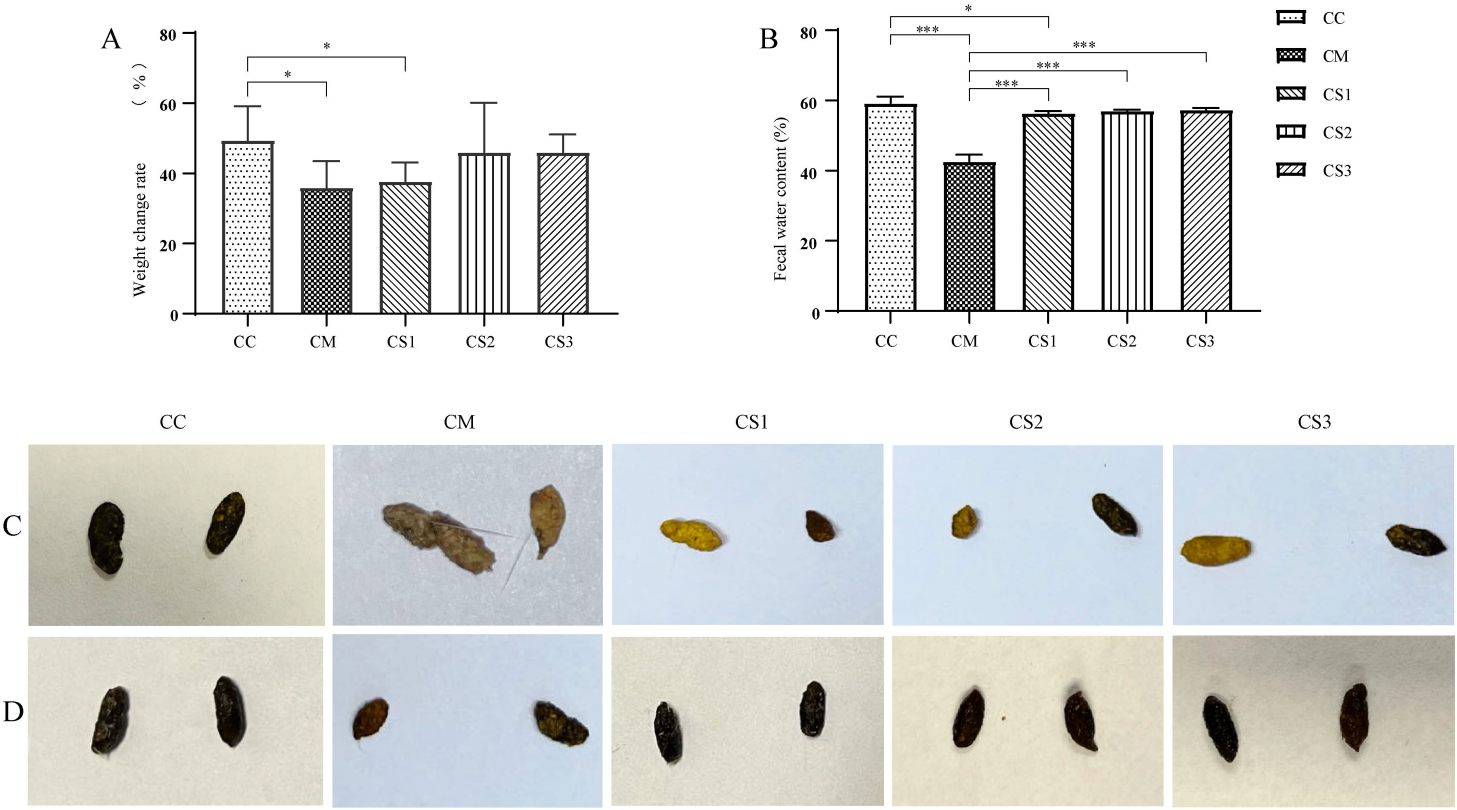
Body weight and fecal characteristics of mice in each group. **(A)** Body weight after MMF intervention. **(B)** Fecal water content after the intervention. **(C)** Fecal morphology after modeling. **(D)** Fecal morphology after MMF treatment. *p< 0.05, ***p< 0.001.

As shown in **Figure 2A**, after the intervention with the three formulations of MMF, the body weights of the CS2 and CS3 groups were not significantly different from those in the CC group (*p* > 0.05), but the body weights of the CM and CS1 groups remained significantly lower than those in the CC group (both *p*< 0.05). **Figure 2B** shows that after MMF intervention, there was no significant difference in fecal water content between the CC, CS2, and CS3 groups (*p* > 0.05), while the fecal water content in the CM and CS1 groups was significantly lower than that in the CC group (*p*< 0.001; *p*< 0.05). Fecal water contents in the CS1, CS2, and CS3 groups were significantly higher than that in the CM group (both *p*< 0.001). **Figure 2C** shows the fecal morphology after modeling, where CC group feces were rice-grain-shaped, smooth, and of moderate consistency. In contrast, feces from the CM, CS1, CS2, and CS3 groups were small, irregular, and hard. **Figure 2D** shows fecal morphology after MMF treatment, where feces from the CC, CS1, CS2, and CS3 groups returned to a black, rice-grain shape, with a smooth appearance and moderate hardness. The CM group still had small, irregular feces. These results indicate that all three formulations of MMF improved body weight and fecal water content in mice, with S1 being the least effective.

### 3.3 Effects of Massa Medicata Fermentata with different formulations on intestinal enzyme activity in mice with spleen deficiency constipation

**Figure 3:**
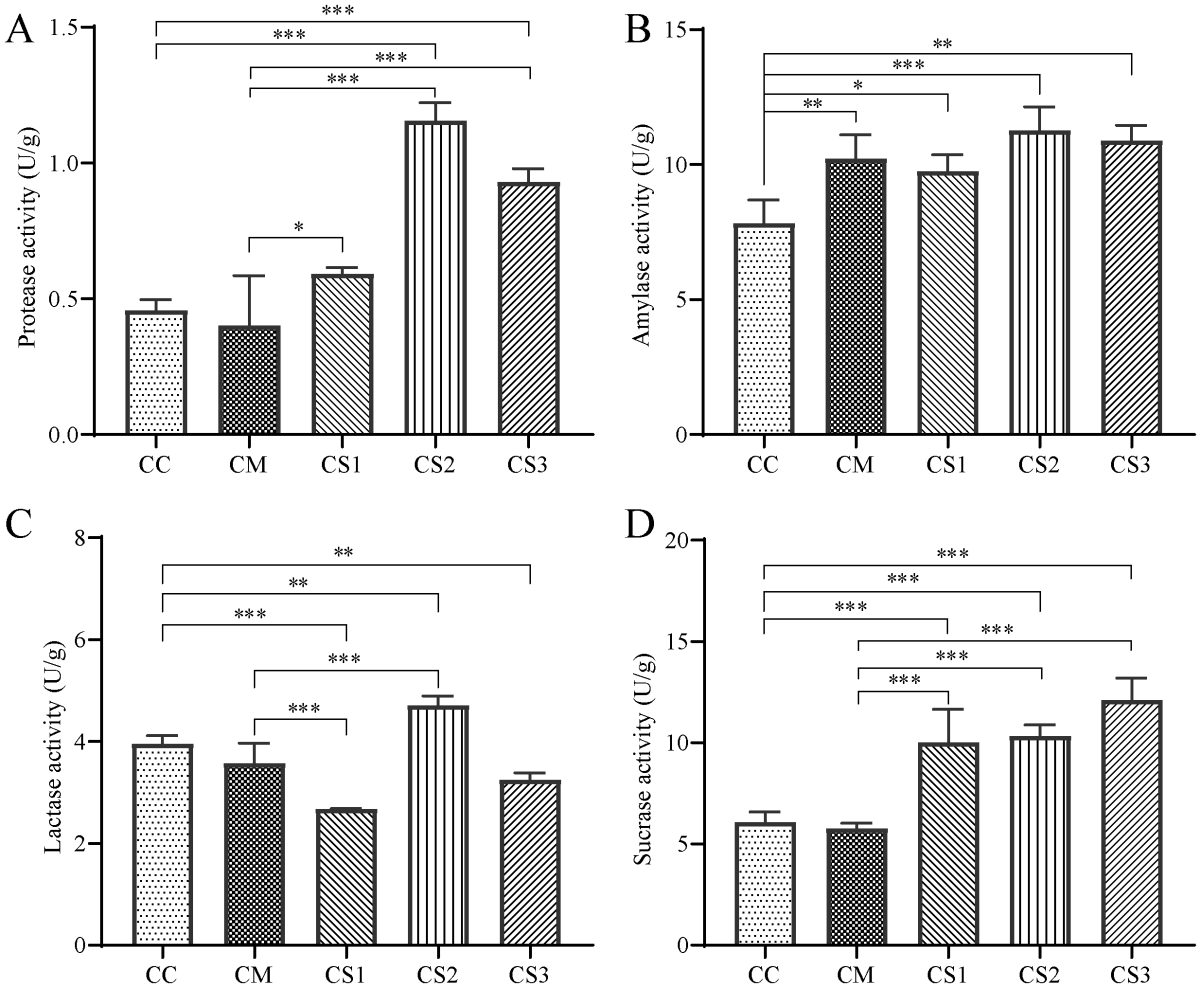
Effects of MMF Ingredients on intestinal enzyme activities in mice with spleen deficiency constipation. **(A)** Protease activity. **(B)** Amylase activity. **(C)** Lactase activity. **(D)** Sucrase activity. *p< 0.05, **p< 0.01, ***p< 0.001.

From the analysis of intestinal protease activity (**Figure 3A**), the CM group showed a slight decrease compared to the CC group (*p* > 0.05), while the CS1 group exhibited a slight increase (*p* > 0.05). Both the CS2 and CS3 groups displayed significantly higher protease activity compared to the CC group (*p*< 0.001). Furthermore, compared to the CM group, the protease activity in the CS1, CS2, and CS3 groups increased significantly (*p*< 0.05, *p<* 0.001, *p<* 0.001), with the CS2 group showing the highest activity. This indicates that spleen deficiency constipation reduces intestinal protease activity in mice, while all three formulations of MMF can enhance protease activity.

Regarding amylase activity (**Figure 3B**), the CM, CS1, CS2, and CS3 groups all showed a significant increase compared to the CC group (*p*< 0.01, *p<* 0.05, *p<* 0.001, *p*< 0.01), with the CS2 group showing the greatest increase. This suggests that the modeling method may lead to elevated intestinal amylase activity in mice, potentially due to the low-fiber rice diet.

For intestinal lactase activity (**Figure 3C**), compared to the CC group, the CM group showed a slight decrease (*p* > 0.05), while the CS1 and CS3 groups exhibited significant decreases (*p<* 0.001, *p*< 0.01). In contrast, the CS2 group showed a significant increase (*p*< 0.01). Lactase activity in the CS1 group was significantly lower than that of the CM group (*p*< 0.01), while lactase activity in the CS2 group was significantly higher than in the CM group (*p*< 0.01).

Regarding intestinal sucrase activity (**Figure 3D**), the CM group showed a slight decrease compared to the CC group, while the sucrase activity in the CS1, CS2, and CS3 groups increased significantly (*p*<0.001, *p*<0.001, *p*<0.001). Compared to the CM group, the sucrase activity in the CS1, CS2, and CS3 groups was also significantly higher (*p*<0.001, *p*<0.001, *p*<0.001). This suggests that all three formulations of MMF can enhance intestinal sucrase activity in mice.

In spleen deficiency constipation mice, the activities of protease, lactase, and sucrase enzymes tend to decrease (*p* > 0.05). MMF S2 and S3 significantly increased protease and sucrase enzymes levels compared with CM group.

### 3.4 Effects of Massa Medicata Fermentata with different formulations on serum D-xylose, VIP, and 5-HT levels in mice with spleen deficiency constipation

**Figure 4:**
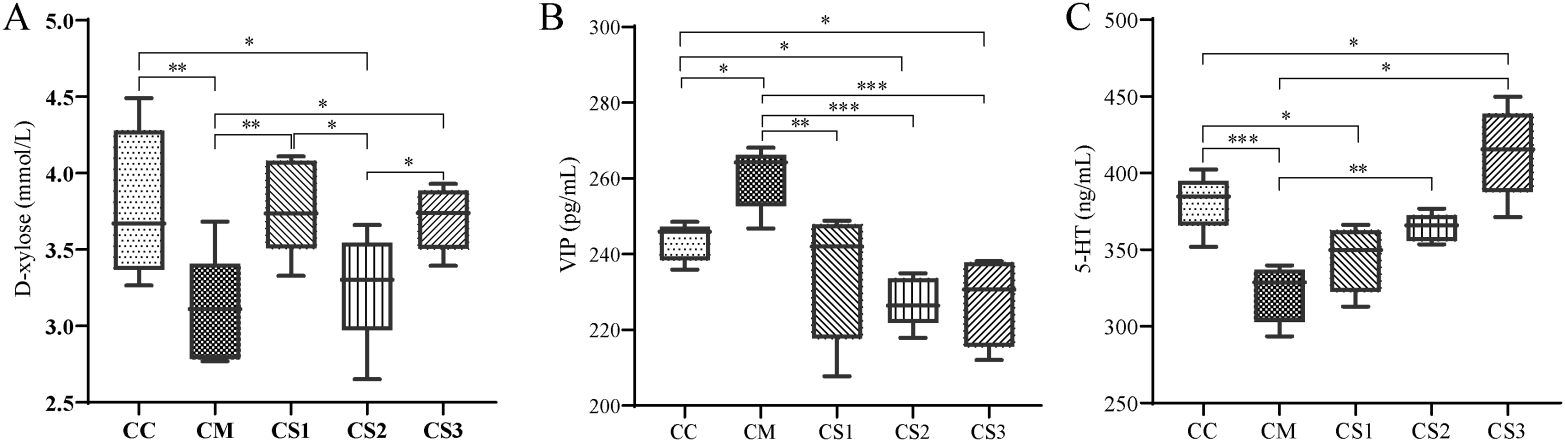
Effects of MMF Ingredients on serum D-xylose, VIP, and 5-HT levels. **(A)** D-xylose levels; **(B)** VIP levels; **(C)** 5-HT levels. *p< 0.05, **p< 0.01, ***p< 0.001.

D-xylose is an important objective indicator for diagnosing spleen deficiency. As shown in **Figure 4A**, compared to the CC group, serum D-xylose levels significantly decreased in the CM and CS2 groups (*p*<0.01, *p*<0.05). Compared to the CM group, serum D-xylose levels significantly increased in the CS1 and CS3 groups (*p*<0.01, *p*<0.05). Compared to the CS2 group, serum D-xylose levels significantly increased in the CS1 and CS3 groups (*p*< 0.05, *p*< 0.05). These findings indicate that the CM and CS2 groups were in a state of spleen deficiency, while MMF S1 and S3 effectively improved spleen deficiency.

VIP is an inhibitory neurotransmitter in the enteric nervous system. Elevated VIP levels can inhibit gastrointestinal smooth muscle contraction, reduce glandular secretion, and slow intestinal motility, leading to chronic constipation. As shown in **Figure 4B**, serum VIP levels significantly increased in the CM group compared to the CC group (*p*< 0.05), while they significantly decreased in the CS2 and CS3 groups (*p*< 0.05, *p*< 0.05). Compared to the CM group, serum VIP levels significantly decreased in the CS1, CS2, and CS3 groups (*p*< 0.01, *p*< 0.001, *p*< 0.001).

Low 5-HT levels weaken intestinal motility and stimulate constipation. **Figure 4C** shows that serum 5-HT levels significantly decreased in the CM and CS1 groups compared to the CC group (*p*<0.001, *p*<0.05), while 5-HT levels significantly increased in the CS3 group (*p*< 0.05). No significant differences were observed between the CS2 and CC groups. Compared to the CM group, 5-HT levels significantly increased in the CS2 and CS3 groups (*p*<0.01, *p*<0.05). These results suggest that VIP and 5-HT are abnormally regulated in mice with spleen deficiency constipation, and the different formulations of MMF could regulate these neurotransmitters to varying degrees.

### 3.5 Effects of Massa Medicata Fermentata with different formulations on the species diversity of intestinal microbiota in mice with spleen deficiency constipation

**Figure 5:**
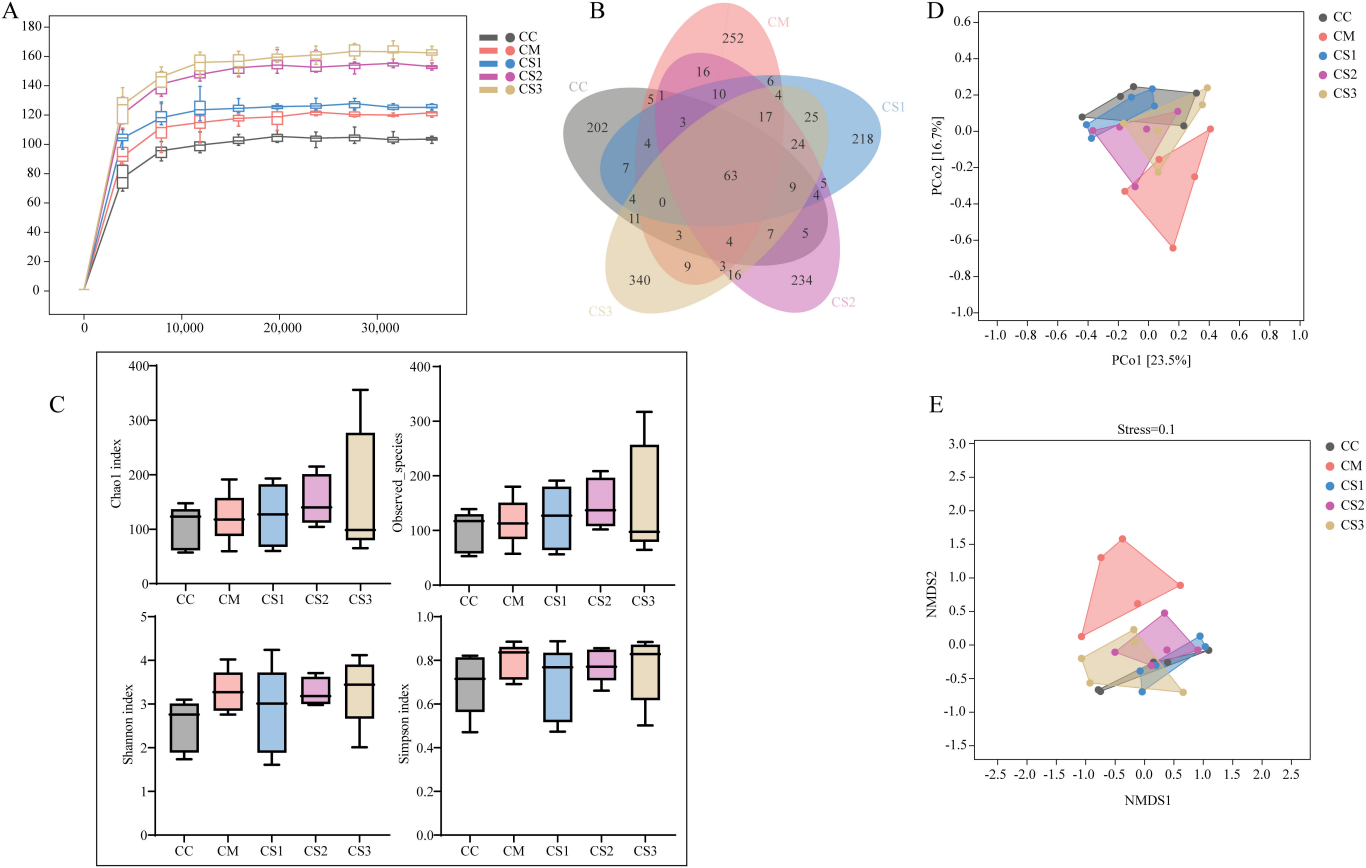
Effects of MMF Ingredients on intestinal microbiota species diversity in mice with spleen deficiency constipation. **(A)** Chao1 rarefaction curve. **(B)** ASV petal diagram. **(C)** Alpha diversity indices. **(D)** PCoA analysis. **(E)** NMDS analysis.

The Chao1 rarefaction curve (**Figure 5A**) reflects the effect of sequencing depth on observed sample diversity. As sequencing depth increased, the curve reached a plateau, indicating that the sequencing depth was sufficient to capture the diversity within the current samples, and additional sequencing would not reveal many undiscovered ASVs. As shown in **Figure 5B**, the total number of ASVs shared by the CC, CM, CS1, CS2, and CS3 groups was 63, while the unique ASVs for each group were 202, 252, 218, 234, and 340, respectively. Compared to the CC group, the total number of ASVs and unique ASVs increased in the CM group. Compared to the CM group, the total number and unique ASVs decreased in the CS1 and CS2 groups, with the CS1 group showing the greatest reduction.

Alpha diversity represents species diversity within a habitat. Chao1 and Observed Species indices reflect species richness, while Shannon and Simpson indices reflect species diversity. As shown in **Figure 5C**, there were no significant differences in Chao1, Observed Species, Shannon, or Simpson indices between the five groups (all *p* > 0.05). Beta diversity measures species composition differences between different environments. The results of PCoA align with the characteristics of ecological data. PCoA projects the sample distance matrix into a low-dimensional space while preserving the original distance relationships between the samples to the greatest extent possible. PCoA results (**Figure 5D**) showed that PC1 contributed 23.5% and PC2 contributed 16.7%. The CC and CM groups had no overlap and were far apart, while the CC group overlapped with the CS1, CS2, and CS3 groups, particularly with the CS1 group. The NMDS results (**Figure 5E**) were consistent with the PCoA results, with a Stress value of 0.1, indicating reliable NMDS analysis. These findings suggest that all three formulations of MMF effectively restored the intestinal microbiota composition in mice with spleen deficiency constipation, with S1 showing the best effect.

### 3.6 Effects of Massa Medicata Fermentata with different formulations on the composition and abundance of dominant intestinal microbiota in mice with spleen deficiency constipation

**Figure 6:**
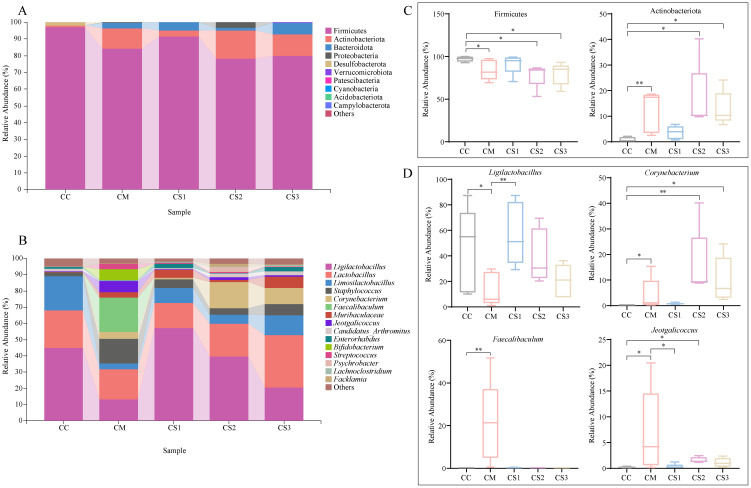
Effects of MMF Ingredients on the composition and abundance of dominant intestinal microbiota in mice with spleen deficiency constipation. **(A)** Relative abundance at the phylum level. **(B)** Relative abundance at the genus level. **(C)** Differential dominant phyla. **(D)** Differential dominant genera. *p< 0.05, **p< 0.01.

The relative abundance of the top 10 dominant phyla and the top 15 dominant genera was visualized in bar plots. As shown in **Figure 6A**, Firmicutes was the dominant phylum in the intestinal microbiota of all groups, with relative abundances of 97.04%, 84.05%, 91.38%, 78.31%, and 79.77% in the CC, CM, CS1, CS2, and CS3 groups, respectively. The second most dominant phylum was Actinobacteriota, with relative abundances of 0.68%, 12.27%, 3.65%, 16.81%, and 12.98%, respectively. At the genus level (**Figure 6B**), *Ligilactobacillus* was the dominant genus, with relative abundances of 45.07%, 13.41%, 56.98%, 39.77%, and 20.50% in the CC, CM, CS1, CS2 and CS3 groups, respectively. The second most abundant genus was *Lactobacillus*, with relative abundances of 22.84%, 18.02%, 15.78%, 20.13%, and 32.22%, respectively. The third most abundant genus was *Limosilactobacillus*, with relative abundances of 21.11%, 3.73%, 9.25%, 5.61%, and 12.35%, respectively. Notably, the relative abundance of *Faecalibaculum* was higher in the CM group, accounting for 21.12%. Statistical analysis of the relative abundance of the top 10 dominant phyla and the top 15 dominant genera (**Figures 6C, D**) revealed that the relative abundance of Firmicutes was not significantly different between the CS1 and CC groups (*p* > 0.05), while the relative abundance of Firmicutes in the CM, CS2, and CS3 groups was significantly lower than that in the CC group (all *p*< 0.05). The relative abundance of Actinobacteriota was not significantly different between the CS1 and CC groups (*p* > 0.05), but it was significantly higher in the CM, CS2, and CS3 groups compared to the CC group (*p*< 0.01; *p*< 0.05; *p*< 0.05). At the genus level, the relative abundance of *Ligilactobacillus* was significantly increased in CC and CS1 group compared with CM group (*p*<0.05, *p*<0.01), but it was not significantly different between the CS1 and CC groups (*p* > 0.05). The relative abundance of *Corynebacterium* was significantly higher in the CM, CS2, and CS3 groups compared to the CC group (*p*< 0.05; *p*< 0.01; *p*< 0.05), but it was not significantly different between the CC and CS1 groups (*p* > 0.05). The relative abundance of *Faecalibaculum* showed significant changes between the CM group compared to the CC group (*p*< 0.01), while it not significantly increased in the CS1, CS2, and CS3 groups (both *p* > 0.05). For *Jeotgalicoccus*, but it was significantly higher in the CM and CS2 group (*p*< 0.05, *p*< 0.05), while the CS1 group showed a significant decrease compared to the CM group (*p*< 0.05), no significant changes were observed between the CS1 and CS3 groups compared to the CC group (*p* > 0.05). These results suggest that spleen deficiency constipation caused significant changes in intestinal microbiota at both the phylum and genus levels, and three formulations of MMF restored the relative abundance of intestinal microbiota to normal levels to a certain extent, with CS1 showing more similar to that of the CC group, the CS2 and CS3 groups performed more closely.

### 3.7 Effects of Massa Medicata Fermentata with different formulations on the characteristic intestinal microbiota in mice with spleen deficiency constipation

**Figure 7:**
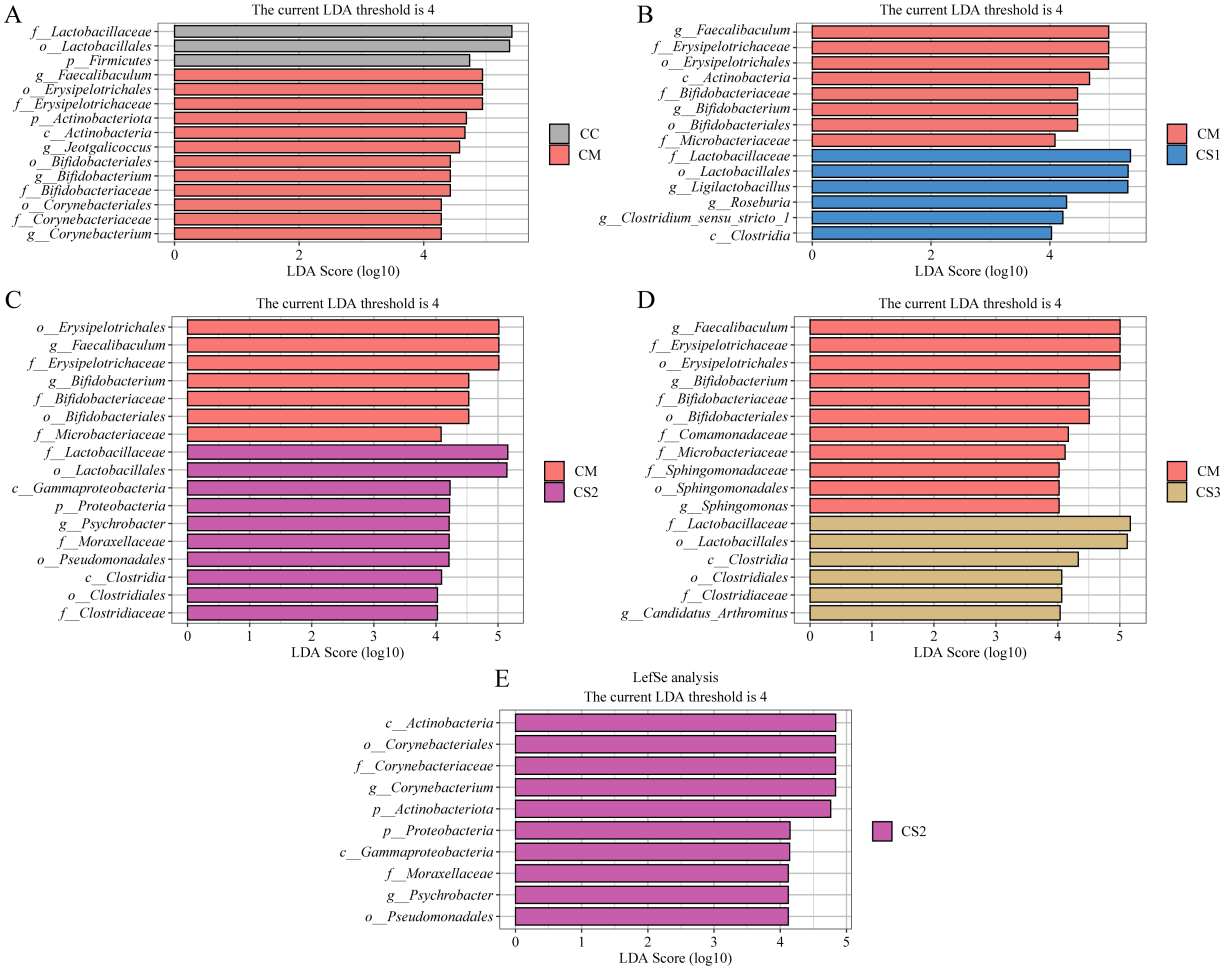
Effects of MMF Ingredients on the characteristic intestinal microbiota in mice with spleen deficiency constipation. **(A)** LEfSe analysis between CC and CM groups. **(B)** LEfSe analysis between CM and CS1 groups. **(C)** LEfSe analysis between CM and CS2 groups. **(D)** LEfSe analysis between CM and CS3 groups. **(E)** LEfSe analysis between CS1、CS2 and CS3 groups.

LEfSe (Linear Discriminant Analysis Effect Size) is a differential analysis method that allows for simultaneous analysis across all taxonomic levels, emphasizing the identification of robust differential species, or biomarkers, between groups. In this study, LEfSe analysis was performed with an LDA threshold of >4.0 to identify characteristic bacterial genera between groups. The LDA value distribution bar plot (**Figure 7**) presents the results of the LEfSe analysis, focusing on the characteristic microbiota between the CC and CM groups, as well as the CM group compared to the CS1, CS2, and CS3 groups, and the comparison among the three MMF groups. As shown in **Figure 7A**, LEfSe analysis between the CC and CM groups identified *Faecalibaculum*, *Jeotgalicoccus*, *Bifidobacterium*, and *Corynebacterium* as the characteristic genera in the CM group. In the LEfSe analysis between the CM and CS1 groups (**Figure 7B**), *Faecalibaculum* and *Bifidobacterium* were identified as characteristic genera in the CM group, while *Ligilactobacillus*, *Roseburia*, and *Clostridium_sensu_stricto_1* were the characteristic genera in the CS1 group. LEfSe analysis between the CM and CS2 groups (**Figure 7C**) identified *Faecalibaculum* and *Bifidobacterium* as characteristic genera in the CM group, and *Psychrobacter* as the characteristic genus in the CS2 group. Finally, LEfSe analysis between the CM and CS3 groups (**Figure 7D**) identified *Faecalibaculum*, *Bifidobacterium*, and *Sphingomonas* as characteristic genera in the CM group, while *Candidatus_Arthromitus* was the characteristic genus in the CS3 group. As shown in **Figure 7E**, *Corynebacterium* and *Psychrobacter* were identified as characteristic genera of the CS2 group. These results indicate that *Faecalibaculum*, *Jeotgalicoccus*, *Bifidobacterium*, *Corynebacterium*, and *Sphingomonas* may serve as potential biomarkers for spleen deficiency constipation. The three formulations of MMF may exert their effects by enriching different characteristic microbiota to improve spleen deficiency constipation.

### 3.8 Effects of Massa Medicata Fermentata with different formulations on the functional pathways of intestinal microbiota in mice with spleen deficiency constipation

**Figure 8:**
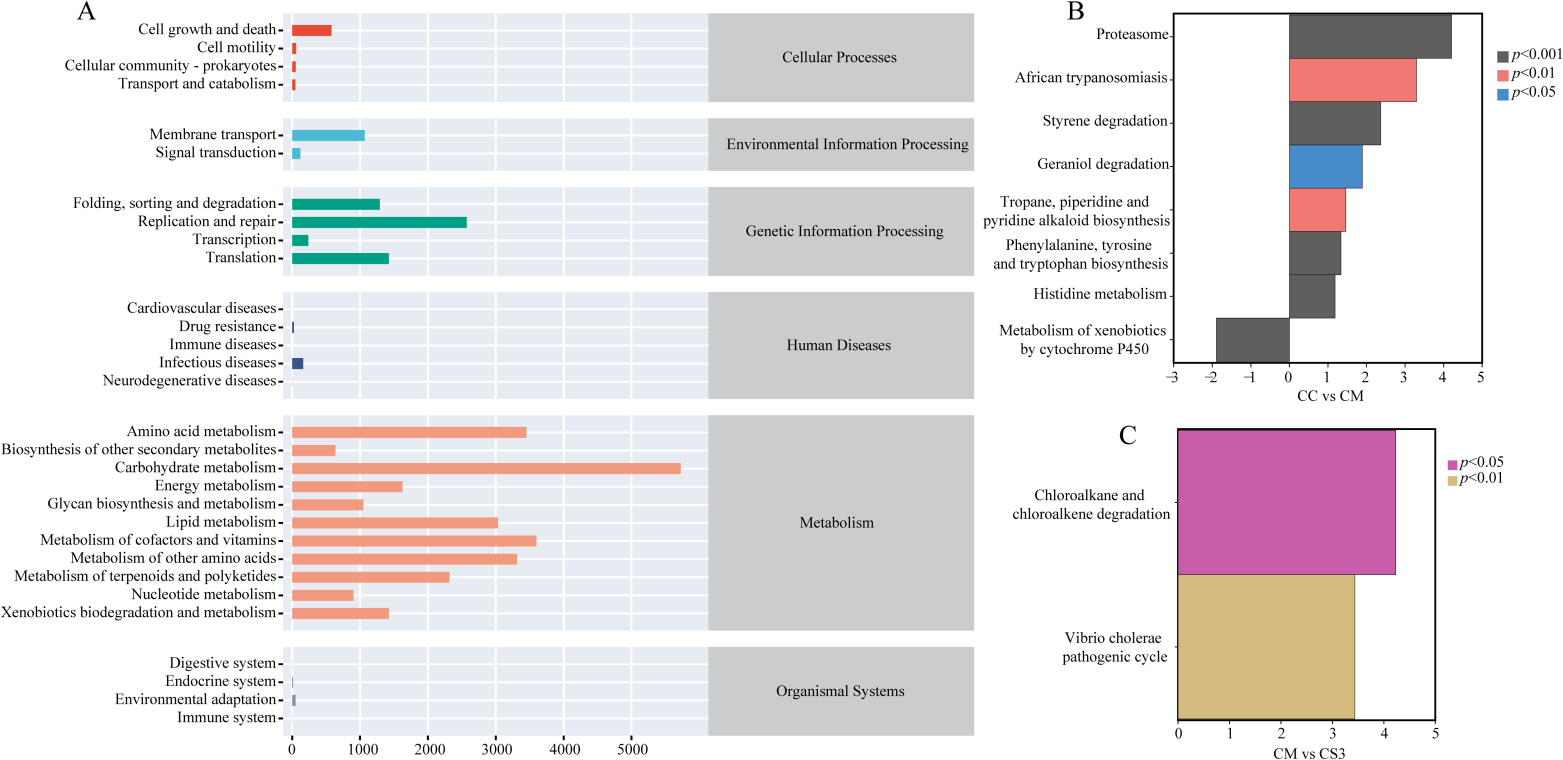
Effects of MMF Ingredients on intestinal microbiota functional pathways in mice with spleen deficiency constipation. **(A)** KEGG metabolic pathways. **(B)** Differential pathways between CC and CM groups. **(C)** Differential pathways between CM and CS3 groups. Positive values on the horizontal axis represent upregulation compared to the control group, while negative values represent downregulation. The vertical axis lists different pathways, and the significance of the differences is indicated by varying colors.

To investigate the effects of spleen deficiency constipation and the three formulations of MMF on the metabolic functions of the intestinal microbiota, the PICRUSt2 method was used to predict the metabolic and functional pathways based on 16S rRNA gene sequencing data. The functional pathways were analyzed using the KEGG database. As shown in **Figure 8A**, the six main functional categories identified were Cellular Processes, Environmental Information Processing, Genetic Information Processing, Human Diseases, Metabolism, and Organismal Systems. Among these, Metabolism, Genetic Information Processing, Environmental Information Processing, and Cellular Processes had the highest relative abundance. On the second level, 30 sub-functional categories were identified. In the Cellular Processes category, cell growth and death had the highest abundance. In the Environmental Information Processing category, membrane transport was most abundant. In the Genetic Information Processing category, replication and repair, were the most abundant. In the Metabolism category, carbohydrate metabolism had the highest abundance. Further analysis (**Figures 8B, C**) revealed that the CM group had significantly downregulated the metabolism of xenobiotics by cytochrome P450 pathway compared to the CC group (*p*< 0.001), while it had significantly upregulated the geraniol degradation pathway compared to the CC group (*p*< 0.05). The CM group also significantly upregulated the african trypanosomiasis and tropane, piperidine, and pyridine alkaloid biosynthesis pathways compared to the CC group (both *p*< 0.01). Additionally, the CM group upregulated the proteasome, styrene degradation, histidine metabolism, and phenylalanine, tyrosine, and tryptophan biosynthesis pathways compared to the CC group (*p*< 0.001). The CS3 group significantly upregulated the chloroalkane and chloroalkene degradation and vibrio cholerae pathogenic cycle pathways compared to the CM group (*p*< 0.05; *p*< 0.01). These results indicate that spleen deficiency constipation affects the functional pathways of intestinal microbiota, and MMF may improve intestinal function by modulating metabolic pathways.

### 3.9 Correlation between characteristic intestinal microbiota and D-xylose, VIP, and 5-HT levels in mice with spleen deficiency constipation

**Figure 9:**
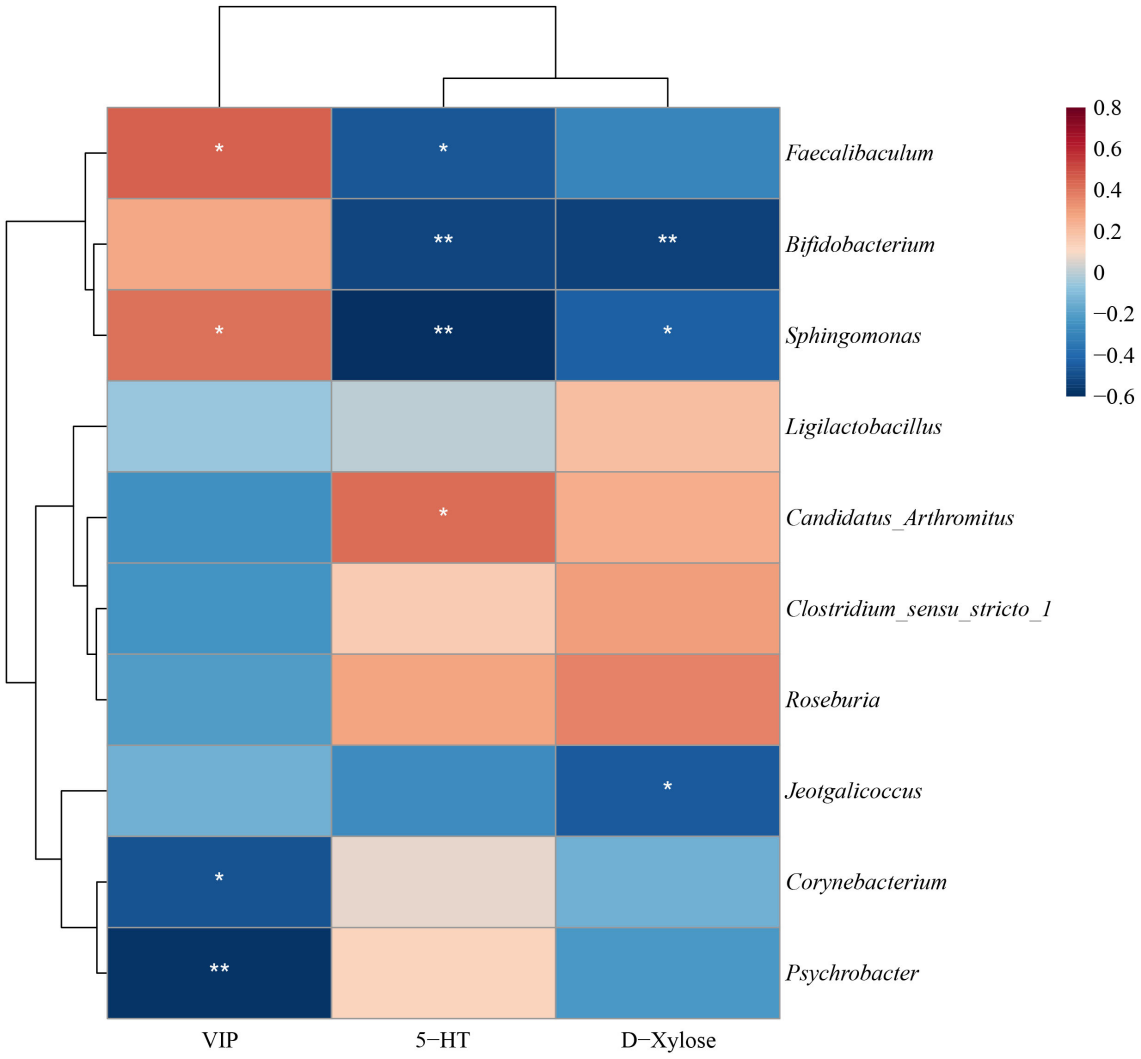
Correlation analysis between characteristic intestinal microbiota and VIP, 5-HT and D-xylose levels. *p< 0.05; **p< 0.01.

LEfSe analysis identified *Faecalibaculum*, *Jeotgalicoccus*, *Bifidobacterium*, *Corynebacterium*, and *Sphingomonas* as characteristic bacterial genera in the CM group, *Ligilactobacillus*, *Roseburia*, and *Clostridium_sensu_stricto_1* in the CS1 group, *Psychrobacter* in the CS2 group, and *Candidatus_Arthromitus* in the CS3 group. To explore the relationship between these characteristic bacteria and physiological indicators (D-xylose, VIP, and 5-HT), Spearman correlation analysis was performed. As shown in **Figure 9**, different characteristic microbiota were correlated with these physiological indicators, with red indicating positive correlations and blue indicating negative correlations, while color intensity reflects the strength of the correlation. D-xylose was significantly negatively correlated with *Sphingomonas*, *Jeotgalicoccus*, and *Bifidobacterium* (*p*< 0.05, *p*< 0.05, *p*< 0.01). 5-HT was significantly negatively correlated with *Faecalibaculum*, *Bifidobacterium*, and *Sphingomonas* (*p*< 0.05, *p*< 0.01, *p*< 0.01), and positively correlated with *Candidatus_Arthromitus* (*p*< 0.05). VIP was significantly positively correlated with *Faecalibaculum* and *Sphingomonas* (both *p*< 0.05), and significantly negatively correlated with *Corynebacterium* and *Psychrobacter* (*p*< 0.05, *p*< 0.01). These results suggest that different characteristic microbiota may play key roles in regulating physiological indicators associated with spleen deficiency constipation, particularly *Candidatus_Arthromitus* in the CS3 group and *Psychrobacter* in the CS2 group, which were positive and negative correlations with 5-HT and VIP, respectively. These may have positive and negative effects on neurotransmitter regulation and alleviation of constipation symptoms.

## 4 Discussion

### 4.1 Massa Medicata Fermentata may improve spleen deficiency constipation by regulating intestinal enzyme activities

MMF contains digestive enzymes, volatile oils, glycosides, ergosterol, flavonoids, B-complex vitamins, and other active components. During the fermentation process, enzymes such as amylase, protease, lipase, cellulase, and glucoamylase are produced, which aid in the breakdown of food and help restore gastrointestinal function (Yin et al., 2021; Yang T. et al., 2023). The intestines naturally contain a large number of digestive enzymes, including amylase, protease, cellulase, and xylanase, which play key roles in digestion (Zhu et al., 2022; Zhou et al., 2023). Among these, cellulase and xylanase are primarily secreted by intestinal microbes, and changes in their activity reflect corresponding changes in intestinal microbiota. Amylase and protease, which are secreted by the host, are important intestinal enzymes, and reductions in their activity can impair digestion and absorption (Zhu et al., 2022).

Research has shown that spleen deficiency constipation leads to significant changes in intestinal microbes and enzymes (Long et al., 2017). In this study, amylase activity significantly increased in mice with spleen deficiency constipation and after MMF intervention. Previous studies have also observed increased amylase activity after spleen deficiency modeling (Long et al., 2017; Zhu et al., 2022), consistent with our findings. This may be related to the low-fiber rice diet used during the modeling stage. Except for amylase, we did not find significant differences in the levels of the other three enzymes between the CM and CC groups. It may be the result of natural recovery of the mice later in the experiment. However, after treatment with MMF, compared with the CM group, the levels of the protease and sucrase enzymes were increased to varying degrees. Overall, this study confirms that the different formulations of MMF can significantly regulate intestinal enzyme activities in mice with spleen deficiency constipation, with S2 and S3 showing prominent effects. These results suggest that MMF plays an important role in treating spleen deficiency constipation by modulating intestinal enzyme activities.

### 4.2 Regulation of intestinal microbiota is an important mechanism for Massa Medicata Fermentata in treating spleen deficiency constipation

Intestinal microbiota is closely related to human health, participating in digestion, metabolism, and inhibiting the growth of pathogenic microorganisms. It also acts as a natural immune barrier, regulating innate immunity and the mucosal barrier of the intestines. Disruption of intestinal microbiota homeostasis can lead to various gastrointestinal diseases, including constipation, irritable bowel syndrome, and inflammatory bowel diseases. In this study, 16S rRNA high-throughput sequencing was used to analyze changes in intestinal microbiota in the small intestines of mice. ASV abundance and species diversity analyses showed that spleen deficiency constipation altered the richness and composition of intestinal microbiota, while different formulations of MMF effectively restored the intestinal microbiota, with S1 showing the best effect, followed by S2 and S3. Both PCoA and NMDS analyses confirmed that S1 had the best effect in restoring intestinal microbiota structure in mice with spleen deficiency constipation.

Based on 16S rRNA sequencing, all three formulations of MMF restored the relative abundance of the dominant phyla, Firmicutes, which decreased in mice with spleen deficiency constipation. A decrease in Firmicutes has been linked to constipation in both Parkinson’s disease and children with autism spectrum disorder (Fu et al., 2021b; DuPont et al., 2023). At the genus level, *Ligilactobacillus* is a lactic acid-producing probiotic that is known for its health benefits (An et al., 2011; Guerrero Sanchez et al., 2022), and *Ligilactobacillus acidipiscis* YJ5 has been shown to alleviate constipation by producing beneficial metabolites and enhancing the intestinal mucosal barrier (Shen et al., 2024). In this study, *Ligilactobacillus* was significantly reduced in spleen deficiency constipation but was restored by all three formulations of MMF, with S1 being the most effective. *Corynebacterium ulcerans* is considered an emerging human pathogen (Hacker et al., 2016), and its abundance significantly increased in mice with spleen deficiency constipation. S1 was the only formulation that restored *Corynebacterium* to normal levels. Although there are few reports on the relationship between *Jeotgalicoccus* and the digestive system, the abundance of *Jeotgalicoccus* has been shown to increase in TGR5 knockout mice, which exhibit heightened anxiety and depression-like behaviors (Tao et al., 2024).

In summary, all three formulations of MMF regulated intestinal microbiota species abundance, diversity, and the relative abundance of dominant bacterial phyla and genera in mice with spleen deficiency constipation.

### 4.3 Characteristic intestinal microbiota are closely related to VIP and 5-HT levels, highlighting their role in the efficacy of Massa Medicata Fermentata

VIP and 5-HT are gastrointestinal hormones that play important roles in regulating gastrointestinal motility. Changes in their levels or receptors can lead to gastrointestinal motility disorders. 5-HT regulates gastrointestinal excitability and inhibition through the enteric nervous system, accelerating colonic motility and transit (Crowell, 2004). Decreased 5-HT secretion reduces smooth muscle contraction and peristalsis, resulting in constipation. In contrast, VIP is an inhibitory gastrointestinal hormone that relaxes gastrointestinal smooth muscles, delays gastric emptying, and reduces gastrointestinal neural excitability. Elevated VIP levels slow intestinal motility, leading to constipation (Cong et al., 2019; Hu et al., 2019). Studies have also shown that Simo Decoction regulates the brain- intestinal axis through intestinal microbiota, influencing VIP and 5-HT levels to promote intestinal health and alleviate constipation (Yi et al., 2023a). Arecoline significantly reduced VIP levels elevated by loperamide, alleviating constipation (You et al., 2020). In this study, spleen deficiency constipation significantly increased VIP levels and decreased 5-HT levels. After intervention with different formulations of MMF, VIP and 5-HT levels were regulated to varying degrees. The effect of S3 seem to be more drastic which significantly reduced VIP and significantly increased 5-HT levels compared with the normal group, while S1 restored VIP to normal levels and S2 normalized 5-HT levels.

Six characteristic bacteria with significant correlation were screened by further correlation analysis between characteristic bacteria and VIP/5-HT, such as *Faecalibaculum*, *Sphingomonas*, *Corynebacterium*, *Psychrobacter*, *Bifidobacterium*, and *Candidatus_Arthromitus*. *Faecalibaculum* was positively correlated with VIP and negatively correlated with 5-HT. This is inconsistent with its report as a beneficial intestinal bacterium, which produces short-chain fatty acids, lowers pH, stimulates intestinal peristalsis, and shortens colonic transit time, thus alleviating constipation (Ma et al., 2023). The reason for this phenomenon may be due to the feedback regulation in the natural recovery process of spleen deficiency constipation mice in the CM group in the later stage. *Sphingomonas* has the same performance as *Faecalibaculum* in the correlation with VIP and 5-HT, it was the characteristic bacteria in the CM group. And the same finding was found in another study on Spleen Deficiency rats treated with Shengmai Yin (You et al., 2020). *Psychrobacter* in the CS2 group was negatively correlated with VIP, it maybe play a laxative role by regulating the content of VIP which revealed that S2 alleviated constipation by regulating the abundance of *Psychrobacter* in intestinal contents in turn affecting the VIP level. Other studies have shown that cinnamic acid effectively treats slow transit constipation by regulating the abundance of bacteria such as *Psychrobacter*, which in turn improves short-chain fatty acid production (Jiang J. et al., 2023). *Candidatus_Arthromitus* has a protective role in the intestinal. and its abundance was found to increase following Zhishe Daozhi Pill intervention in constipation caused by a high-fat, high-protein diet (Peng et al., 2023). Our study further found that it showed a significant positive correlation with 5-HT in the CS3 group, which revealed that S3 alleviated constipation by regulating the abundance of *Candidatus_Arthromitus* in intestinal contents in turn affecting the 5-HT level.

In summary, VIP and 5-HT are important physiological indicators in the development of spleen deficiency constipation. The significant correlations between characteristic intestinal microbiota and the levels of VIP and 5-HT suggest that the interaction between these indicators and the intestinal microbiota may play a key role in the intervention of spleen deficiency constipation by MMF, such as *Psychrobacter* and *Candidatus_Arthromitus* crucially.

## 5 Conclusion

Spleen deficiency constipation is closely related to intestinal microbiota dysbiosis, decreased intestinal enzyme activities, and abnormal VIP and 5-HT levels. The therapeutic effects of the three formulations of MMF on spleen deficiency constipation are achieved by regulating intestinal microbiota, intestinal enzyme activities, and gastrointestinal hormones. Different formulations of MMF have different mechanisms of regulating constipation through intestinal microbiota. MMF S2 alleviated constipation by regulating the abundance of *Psychrobacter* in intestinal contents in turn affecting the VIP level, while S3 by regulating the abundance of *Candidatus_Arthromitus* in turn affecting the 5-HT level. Although this study revealed the multifaceted effects of MMF in treating spleen deficiency constipation, further research is needed to elucidate the specific pathways and signaling mechanisms involved in regulating intestinal microbiota, enzyme activities, and gastrointestinal hormones.

## Data Availability

The datasets presented in this study can be found in online repositories. The names of the repository/repositories and accession number(s) can be found in the article/supplementary material.
